# Inosine attenuates spontaneous activity in the rat neurogenic bladder through an A_2B_ pathway

**DOI:** 10.1038/srep44416

**Published:** 2017-03-15

**Authors:** Claire Doyle, Vivian Cristofaro, Bryan S. Sack, Stefan N. Lukianov, Mattias Schäfer, Yeun Goo Chung, Maryrose P. Sullivan, Rosalyn M. Adam

**Affiliations:** 1 Urological Diseases Research Center, Boston Children’s Hospital, Boston, MA, USA; 2 Department of Surgery, Harvard Medical School, Boston, MA, USA; 3 Division of Urology, VA Boston Healthcare System, West Roxbury, MA, USA; 4 Department of Surgery, Brigham and Women’s Hospital, Boston, MA, USA

## Abstract

Neurogenic detrusor overactivity (NDO) is among the most challenging complications of spinal cord injury (SCI). A recent report by us demonstrated an improvement in NDO in SCI rats following chronic systemic treatment with the purine nucleoside inosine. The objective of this study was to investigate the mechanism of action of inosine underlying improvement of NDO. Male Sprague-Dawley rats underwent complete spinal cord transection at T8. Inosine (1 mM) delivered intravesically to SCI rats during conscious cystometry significantly decreased the frequency of spontaneous non-voiding contractions. In isolated tissue assays, inosine (1 mM) significantly decreased the amplitude of spontaneous activity (SA) in SCI bladder muscle strips. This effect was prevented by a pan-adenosine receptor antagonist CGS15943, but not by A_1_ or A_3_ receptor antagonists. The A_2A_ antagonist ZM241385 and A_2B_ antagonist PSB603 prevented the effect of inosine. The effect of inosine was mimicked by the adenosine receptor agonist NECA and the A_2B_ receptor agonist BAY60-6583. The inhibition of SA by inosine was not observed in the presence of the BK antagonist, iberiotoxin, but persisted in the presence of K_ATP_ and SK antagonists. These findings demonstrate that inosine acts via an A_2B_ receptor-mediated pathway that impinges on specific potassium channel effectors.

Traumatic injury to the spinal cord, as well as congenital conditions such as spina bifida, can result in diminished urinary tract function. In such patients, the bladder commonly displays reduced capacity, poor compliance and overactivity. In addition detrusor-sphincter dyssynergia (DSD) – a loss of coordination between detrusor smooth muscle contraction and urethral sphincter relaxation – leads to high storage and voiding pressures, and subsequent bladder wall remodelling. Management of neurogenic detrusor overactivity (NDO) in patients with spinal cord injury (SCI) emphasises catheterisation to overcome DSD and achieve bladder emptying, together with medication to reduce non-voiding contractions and improve compliance (reviewed in ref. 1). Pharmacological management of NDO has focused significantly on attenuation of cholinergic signalling, either through anti-muscarinic receptor blockade or by inhibition of neurotransmitter release. Current first-line treatment with oral anti-muscarinic agents is often associated with undesirable systemic side effects that limits its long-term use, and its efficacy has been reported to be variable^2^. Botulinum toxin A has demonstrated significant improvements in a variety of urodynamic parameters^3^ but requires repeat endoscopic administration due to limited durability. Therefore characterisation of alternative agents to manage NDO is merited.

In a recent study, we demonstrated that the purine nucleoside inosine, delivered systemically by daily intraperitoneal administration for six weeks, elicited significant improvements in NDO, as demonstrated by almost complete inhibition of spontaneous non-voiding contractions during cystometry in a rat model of SCI^4^. In that study, chronic administration of inosine was associated with alterations in markers of sensory neurotransmission within the bladder and dorsal root ganglion, consistent with its known neuroprotective and neurotrophic effects (reviewed in ref. 5). However, the signalling mechanisms underlying the ability of inosine to improve NDO were not explored.

In other experimental systems, inosine has been shown to act through adenosine receptors. Signalling via adenosine receptors has been implicated in a wide variety of physiological processes, including pain^6^,^7^ and micturition^8^,^9^,^10^. In a rat model of Parkinson’s disease, intravenous, intrathecal or intracerebroventricular administration of an adenosine A_2A_ receptor antagonist reduced the overactive bladder phenotype^9^ consistent with central and spinal sites of action. In a separate study, intravesical administration of an A_1_ receptor agonist increased the intercontraction interval in rats undergoing cystometry, suggesting involvement of local A_1_ receptor activation in regulation of micturition^10^. Adenosine acting via A_2A_ and A_2B_ receptors has also been implicated in modulation of detrusor contraction and relaxation^11^,^12^. However, neither the role of adenosine receptor signalling in regulating urinary tract function following SCI nor its involvement in the mechanism of action by which inosine improves NDO has been explored.

The aim of this study was to investigate the role of adenosine receptor signalling in the ability of inosine to attenuate detrusor overactivity in the context of SCI, and to explore local action of inosine in the bladder. Intravesical application of anti-muscarinic treatment with oxybutynin has recently demonstrated benefit in patients due to fewer adverse reactions compared to systemic administration^13^,^14^. Therefore intravesical delivery of inosine may also have utility in the context of NDO. Intravesical inosine decreased the frequency and amplitude of non-voiding contractions during conscious cystometry. In addition, inosine decreased spontaneous activity in bladder muscle strips from rats with SCI, and this effect was prevented by adenosine receptor blockade. Among adenosine receptor subtypes, A_2B_ emerged as a dominant mediator of the inhibitory effect of inosine on spontaneous activity. Inosine action on spontaneous activity was also prevented in the presence of an inhibitor of the large conductance potassium channel (BK channel). Taken together, these findings suggest that inosine can act locally within the bladder via the adenosine A_2B_ receptor to inhibit non-voiding contractions and spontaneous activity, and represents a novel treatment for neurogenic detrusor overactivity.

## Results

### Intravesical administration of inosine attenuates NDO in SCI rats

**Figure 1:**
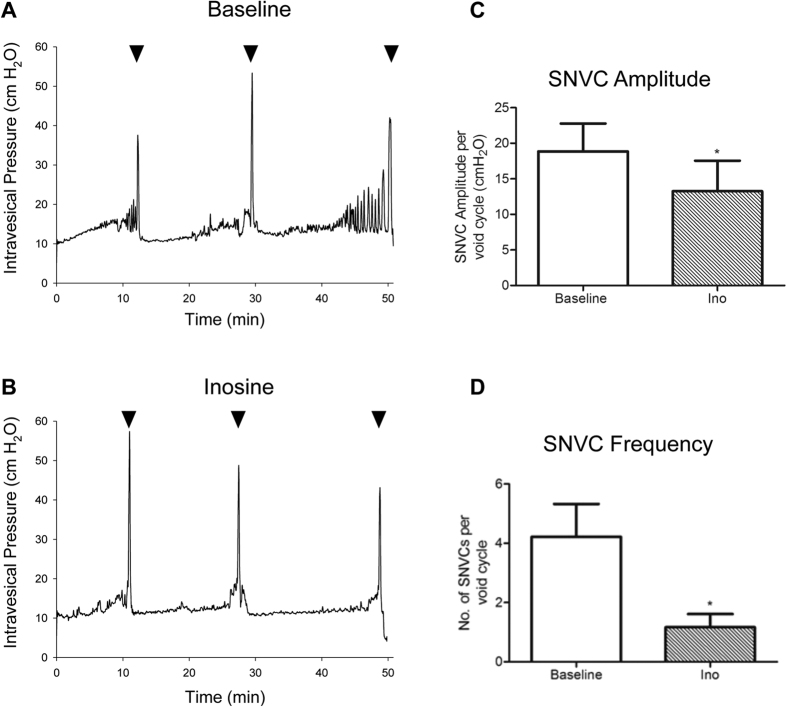
Intravesical inosine attenuates detrusor overactivity following spinal cord injury. (**A**) Representative cystometrograms showing frequent large amplitude SNVCs. (**B**) Intravesical delivery of inosine (1 mM) to the SCI bladder decreased the amplitude and frequency of SNVCs. Arrowheads indicate voids. Time (in min) is indicated on the x-axis. Mechanical artifacts evident in real time were filtered. Inosine decreased the amplitude (**C**) and frequency (**D**) of SNVCs (p < 0.05). Descriptive statistics for different urodynamic parameters are shown in Table 1.

Our previous study demonstrated significant improvements in NDO in SCI rats receiving chronic systemic treatment with inosine^4^. We determined whether inosine could elicit comparable effects when administered locally within the bladder. Rats subjected to spinal cord transection for 6.5 wk underwent conscious cystometry during which inosine (1 mM) was instilled into the bladder. As shown in Fig. 1A, cystometrograms from SCI rats at baseline demonstrated extensive detrusor overactivity, characterised by frequent spontaneous non-voiding contractions (SNVCs). Intravesical delivery of inosine to the bladder led to a decrease in both the amplitude and frequency of SNVCs (Fig. 1B). Summary graphs illustrate a significant decrease in both the amplitude (Fig. 1C) and frequency (Fig. 1D) of SNVCs following inosine administration. Table 1 summarises the effect of inosine on urodynamic parameters in SCI rats. No significant change in compliance during the storage phase was noted. During the voiding phase several parameters were also investigated. A significant decrease in voided volumes and voiding pressure was observed in SCI bladders treated with intravesical inosine. No differences in other voiding parameters such as bladder capacity, peak micturition pressure or micturition interval were noted.

### Inosine decreases spontaneous activity in bladder tissues *in vitro*

**Figure 2:**
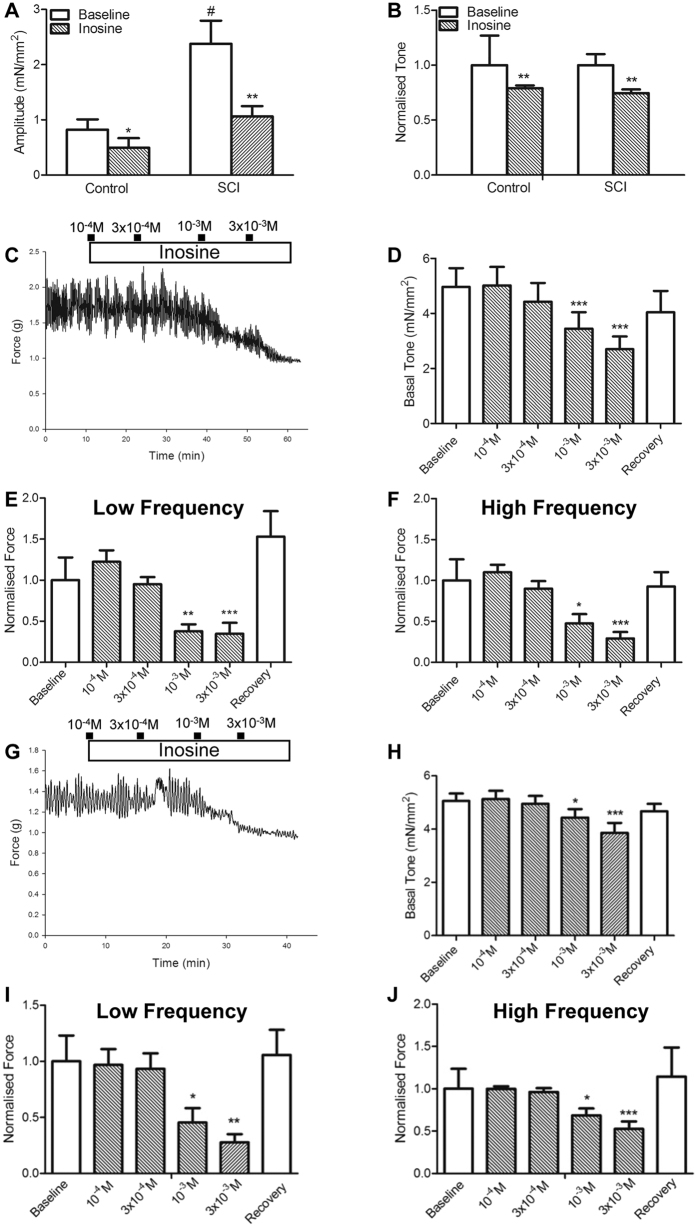
*In vitro* exposure of bladder tissue to inosine decreases spontaneous activity (SA). (**A**) Summary graph demonstrating a significant difference in baseline spontaneous activity between bladder tissues from control (uninjured) and SCI rats (^#^p < 0.05), as well as a significant inhibitory effect of exogenous inosine (1 mM) on spontaneous activity in tissue from control (*p < 0.05, baseline vs. inosine) and SCI (**p < 0.01, baseline vs. inosine) rats. (**B**) Basal tone was also inhibited significantly by inosine in tissue from control and SCI rats (**p < 0.01). (**C**) Representative trace indicating the dose-dependent effect of exogenous inosine when administered to SCI bladder tissue. (**D**) Tone was significantly decreased with increasing doses of inosine (***p < 0.001). (**E,F**) Summary graphs illustrating that inosine (1 mM) decreased the amplitude of both low (**p < 0.01) and high frequency (*p < 0.05, n = 8) components of SA. (**G–J**) Inosine also decreased the tone and amplitude of SA in SCI bladder strips without mucosa. Amplitude data were normalised by baseline responses. Data are presented as mean ± SEM.

Next, we determined the impact of inosine on spontaneous activity (SA) of isolated bladder tissues. The amplitude and frequency of SA that occurred at low and high frequency was determined as illustrated in Supplementary Figure 1A and described in Methods. In the following sections, data in graphs are presented as normalised values (mean ± SEM), except where indicated; raw data are presented in Supplementary Tables 1–5. Initially we compared isometric tension generation in tissues from control (uninjured) and SCI rats. Absolute baseline SA (Fig. 2A) was higher in tissues from SCI rats compared to uninjured controls, consistent with prior observations from our group^15^. In tissues from both control and SCI rats, treatment with inosine led to a significant reduction in both SA and tone (Fig. 2A,B). In tissues from SCI rats the inhibitory effect of inosine on the amplitude of SA was dose-dependent (Fig. 2C). Inosine at 1 mM and 3 mM also significantly decreased the tone of bladder strips (Fig. 2D), which was reversed upon washout. The amplitude of both low and high frequency SA was significantly inhibited by inosine at concentrations of 1 mM and 3 mM, as shown in the summary graphs in Fig. 2E,F. In contrast, inosine did not significantly alter the frequency of either low or high frequency components of SA (Supplementary Figure 1B,C). SCI bladder strips in which the mucosa had been removed also displayed basal spontaneous activity, albeit of lower amplitude (Fig. 2G). As in intact tissue strips, inosine decreased both tone (Fig. 2H) and spontaneous activity in a dose-dependent manner (Fig. 2I,J) but did not alter the mean frequency of low or high frequency SA (Supplementary Figure 1D,E).

### The effect of inosine is mediated via adenosine receptors

**Figure 3:**
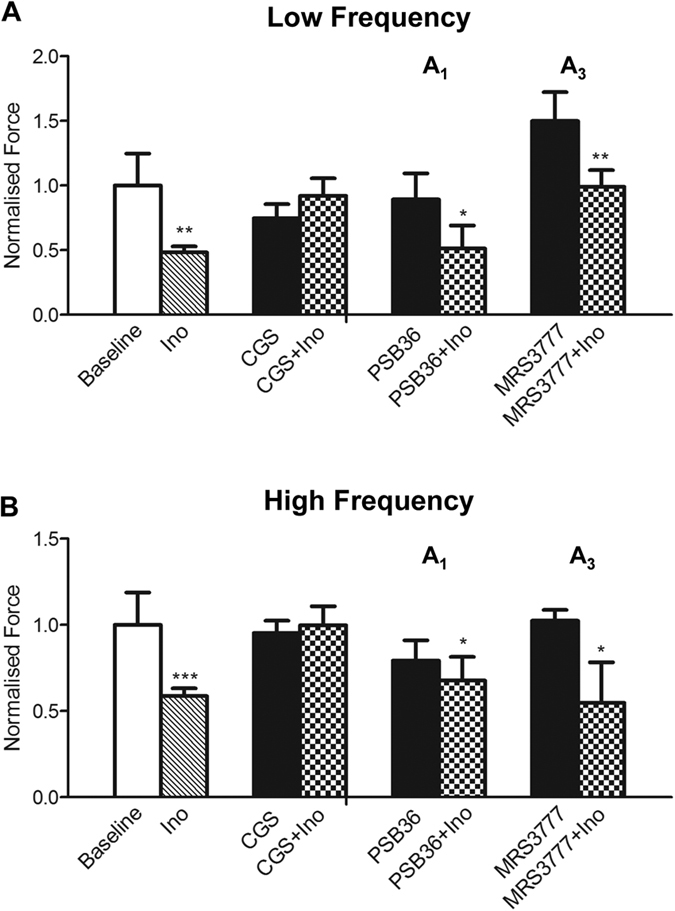
Inosine activity is reversed by adenosine receptor blockade. The amplitude of SA in untreated conditions at both low frequency (**A**) and high frequency (**B**) was significantly reduced by inosine (1 mM; n = 14, **p < 0.01). However, in the presence of a pan-adenosine receptor antagonist CGS15943 (5 μM, n = 7), inosine has no significant effect on SA. In the presence of. the A_1_ receptor antagonist PSB36 (0.1 μM, n = 5) or the A_3_ receptor antagonist MRS3777 (0.1 μM) the effect of inosine on attenuating SA remained significant (PSB36 versus PSB36 + Ino, *p < 0.05; MRS3777 versus MRS3777 + Ino **p < 0.01), excluding A_1_ and A_3_ receptors as mediators of inosine activity. Amplitude data are normalised by baseline responses. Data are expressed as mean ± SEM.

We employed a panel of pharmacological antagonists to determine the role of adenosine receptors in inosine-induced inhibition of SA. For these experiments inosine at a dose of 1 mM was used, and we confirmed that solvents used to prepare antagonists had no effect on SA prior to testing (Supplementary Figure 2). As above, inosine reduced the amplitude of both low (p < 0.01, n = 14, Fig. 3A) and high frequency (p < 0.0001, Fig. 3B) components of spontaneous activity. The mean frequency of SA determined from the low and high frequency components SA was not affected by inosine (p = 0.538, n = 14, Supplementary Figure 3). In the presence of the pan-adenosine receptor antagonist CGS15943 (5 μM), inosine did not reduce the amplitude of SA at low (p = 0.4108, n = 7) or high frequency (p = 0.7872, n = 7). These findings suggest that inosine attenuates spontaneous activity through an adenosine receptor-mediated signalling pathway.

The A_1_ receptor-specific antagonist PSB36 (0.1 μM) did not prevent the ability of inosine to attenuate the amplitude of either low frequency SA (p = 0.0124, n = 5) or high frequency SA (p = 0.0489, n = 5). Similarly, MRS3777 (0.1 μM), a specific antagonist for the A_3_ receptor, did not prevent the effect of inosine on low frequency SA (p = 0.001, n = 5, Fig. 3A) or high frequency SA (p = 0.0474, n = 5, Fig. 3B). Neither CGS15943, PSB36 nor MRS3777 had any effect on the frequency of either low or high frequency SA (Supplementary Figure 3). Thus, inosine-mediated inhibition of the amplitude of SA occurred independently of A_1_ and A_3_ adenosine receptor subtypes, suggesting A_2_ receptors as potential mediators of this response.

### The attenuation of SA by inosine is prevented by A_2A_ and A_2B_ inhibition

**Figure 4:**
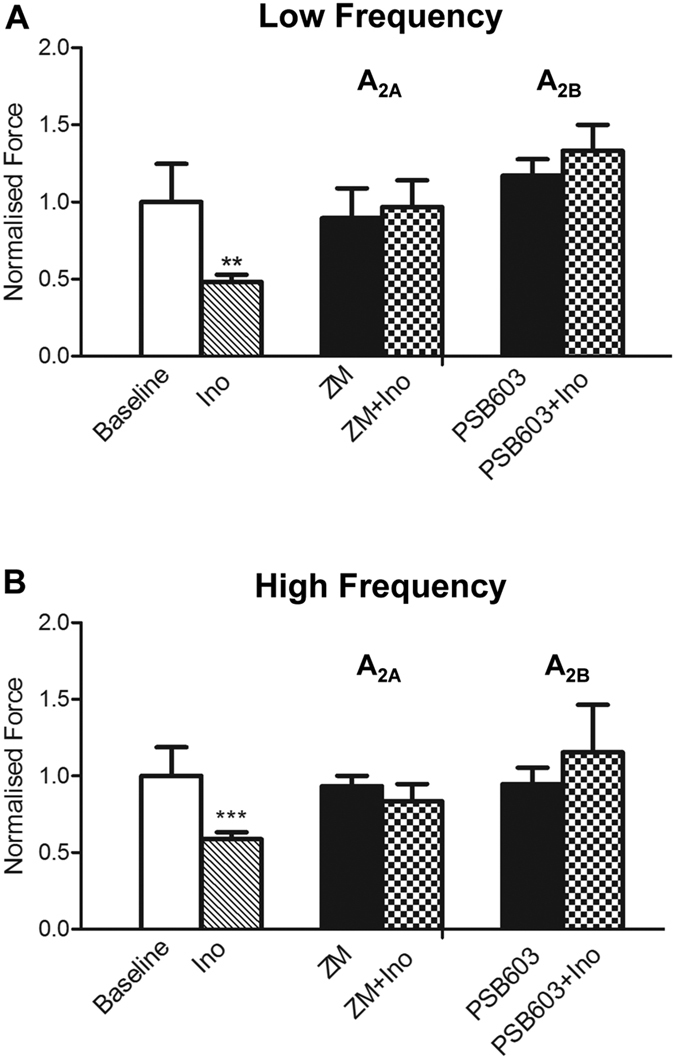
Inosine activity is reversed by A_2A_ and A_2B_ receptor antagonists. (**A**) The amplitude of low frequency activity was significantly attenuated by inosine (1 mM, n = 14, **p < 0.01). Inosine had no effect on SA in the presence of the A_2A_ antagonist ZM241385 (100 μM, n = 7) and the A_2B_ antagonist PSB603 (0.1 μM, n = 7). (**B**) A_2A_ and A_2B_ receptor antagonists prevented the inosine-induced attenuation of the amplitude of high frequency activity. Amplitude data are normalised by baseline responses. Data are expressed as mean ± SEM.

Consistent with a potential role for A_2_ receptors in mediating the effect of inosine, the A_2A_ antagonist ZM241385 (100 μM) inhibited the ability of inosine to attenuate the amplitude of low frequency SA (p = 0.7042, n = 7) (Fig. 4A). Similarly, ZM241385 prevented the inhibitory effect of inosine on the amplitude of high frequency SA (p = 0.4693, n = 7)(Fig. 4B). The frequency of SA at low and high frequencies was not affected by ZM241385 (Supplementary Figure 3). The A_2B_ antagonist PSB603 (0.1 μM) was able to reverse the inhibitory effect of inosine on the amplitude of both low frequency SA (p = 0.1883, n = 7)(Fig. 4A) and high frequency SA (p = 0.4583, n = 7)(Fig. 4B). The frequency of neither low nor high frequency SA was affected in the presence of inosine or PSB603 (Supplementary Figure 3).

**Figure 5:**
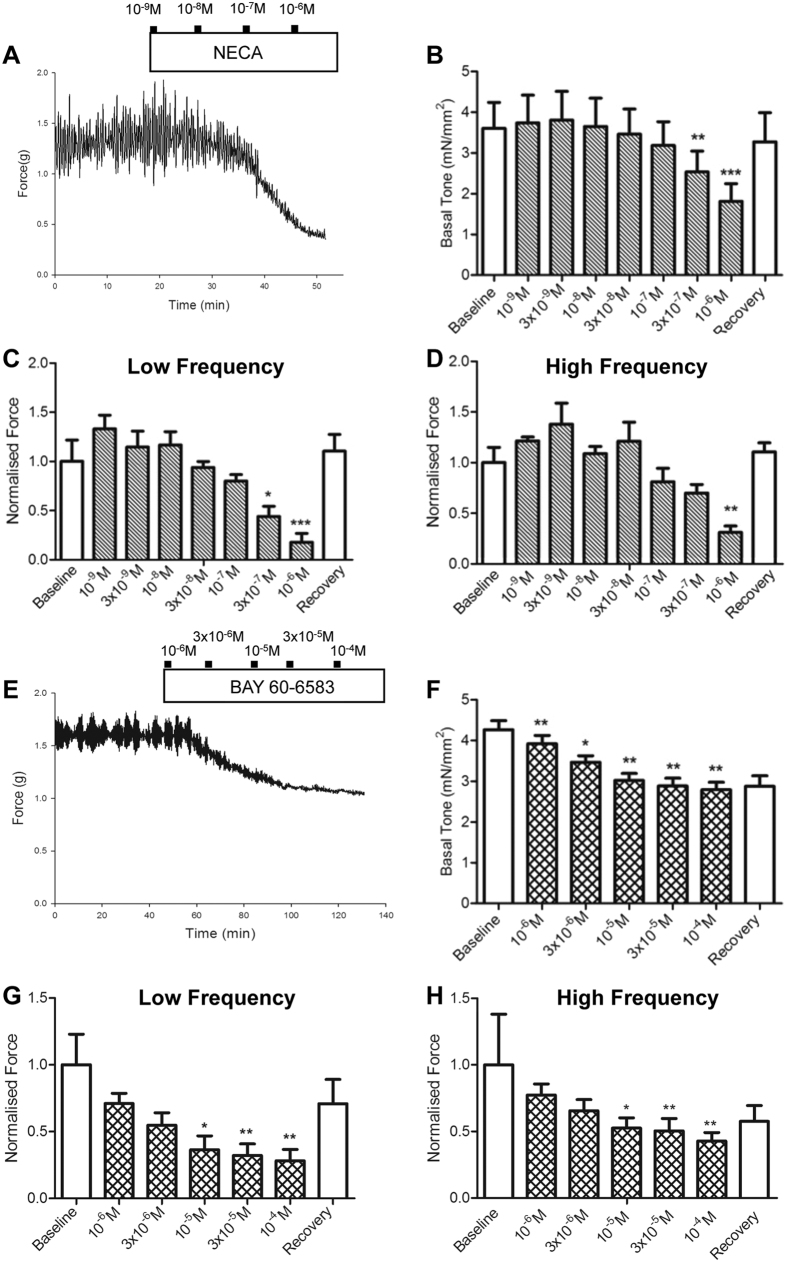
The effect of inosine is mimicked by adenosine receptor agonists. (**A**) Representative trace indicating the dose-dependent effect of the A_2_ agonist NECA (10^−9^M to 10^−6^M, n = 6). (**B**) Tone was significantly decreased with increasing doses of NECA. (**C**,**D**) Summary graphs illustrating a significant decrease in amplitude of low and high frequency SA in the presence of NECA. The average frequency of low and high frequency activity was not affected by NECA. (**E**) Representative trace indicating the dose-dependent effect of the specific A_2B_ agonist BAY60-6583 (10^−6^ M to 10^−4^ M). (**F**) Tone was significantly decreased with increasing doses of BAY60-6583. (**G**,**H**) Summary graphs illustrating a significant decrease of the amplitude of low and high frequency activity in the presence of BAY60-6583 (*p < 0.05 at 10^−5^ M, n = 7). The average frequency of low and high frequency activity was not affected by BAY60-6583. Amplitude data are normalised by baseline responses. Data are represented as mean ± SEM.

Next, we determined whether general and receptor-specific A_2_ agonists could mimic the effect of inosine on SA. The adenosine receptor agonist NECA reduced the tone (Fig. 5A,B) as well as the amplitude of both low and high frequency components of SA (Fig. 5C,D) in a dose-dependent manner. Incubation of bladder strips with the A_2A_ agonist, PSB0777 did not reduce the amplitude of either low or high frequency SA (n = 5, Supplementary Figure 4). In contrast, the potent A_2B_ agonist BAY60-6583 elicited a dose-dependent reduction in both tone (Fig. 5E,F) and SA amplitude (Fig. 5G,H). Summary graphs in Fig. 5G,H demonstrated a significant reduction in the amplitude of both low frequency SA and high frequency SA in tissues exposed to BAY60-6583. Consistent with the activity of inosine, the frequency of SA was not altered by NECA, PSB0777 or BAY60-6583 (Supplementary Figure 4).

To determine whether the relative sensitivity of tissues to A_2A_ and A_2B_ agonists was related to expression level of the receptors, we assessed mRNA levels for A_2A_ and A_2B_. The relative A_2A_ and A_2B_ mRNA levels were comparable in mucosal specimens, whereas in detrusor specimens, relative A_2B_ mRNA levels were higher than those for A_2A_, although there was no difference between tissues from control versus SCI rats (Supplementary Figure 5).

### Inosine elicits its downstream effects via large conductance potassium (BK) channels

**Figure 6:**
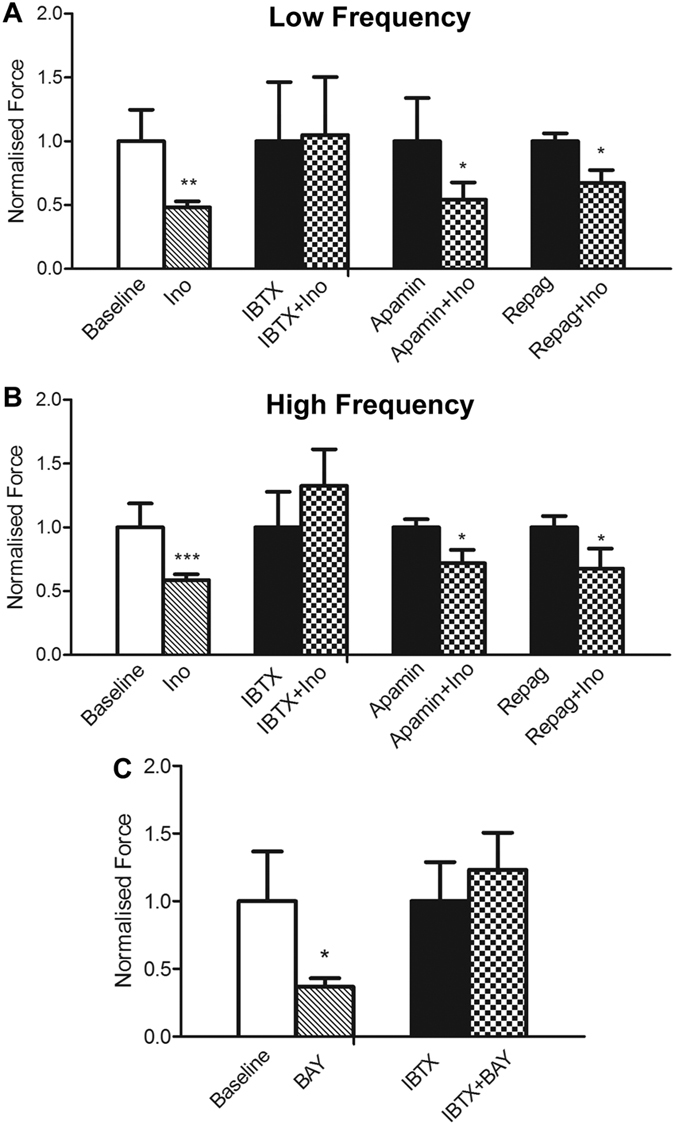
Inosine activity is blocked by BK channel inhibition. (**A**,**B**) The attenuation of SA by inosine was reversed by the large conductance Ca^2+^-activated potassium channel blocker iberiotoxin (IBTX, 100 nM, n = 7). The SK3 channel blocker apamin (100 nM, n = 7) and the K_ATP_ channel blocker repaglinide (10 μM, n = 8) did not prevent the attenuation of SA induced by inosine (**p < 0.01, **p < 0.001, Baseline versus Ino; *p < 0.05, Apamin versus Apamin + Ino, Regap versus Repag + Ino). (**C**) The attenuation of SA by the A_2B_ agonist BAY60-6583 (100 μM) was blocked by IBTX (100 nM, n = 5). Data are expressed as mean ± SEM, but with values normalised to drug alone, as opposed to baseline, as described in Methods.

To connect signalling through adenosine receptors to mediators of the reduction in spontaneous activity in bladder strips, we investigated a role for potassium channels. Activation of potassium channels allows for hyperpolarisation of the cell membrane and relaxation of smooth muscle^16^. The inhibitory activity of inosine on the amplitude of low or high frequency SA was not prevented in the presence of the SK3 small conductance blocker apamin (100 nM) or the K_ATP_ blocker repaglinide (10 μM) suggesting that these channels do not mediate the effect of inosine on SA. In contrast, IBTX (100 nM), an inhibitor of the large conductance BK channel blocked the inhibitory effect of inosine on the amplitude of low frequency SA (p = 0.9235, n = 7, Fig. 6A) and high frequency SA (p = 0.2912, n = 7, Fig. 6B). As anticipated from the known activity of BK channels^16^, IBTX alone increased SA of bladder strips by ~40%. The frequency of low and high frequency SA was not affected by either inosine or IBTX (Supplementary Figure 6). IBTX also blocked the ability of the A_2B_ agonist BAY60-6583 to attenuate SA (Fig. 6C), consistent with a role for A_2B_ receptors as mediators of inosine action.

## Discussion

In this study, we investigated the effects of inosine delivery to the bladder on NDO and explored the underlying signalling events. Our findings demonstrate (i) an improvement in neurogenic detrusor overactivity in SCI rats following intravesical inosine administration as evidenced by a significant reduction in the frequency and amplitude of non-voiding contractions; (ii) reduced spontaneous activity in detrusor muscle strips following acute exposure to inosine; (iii) identification of the A_2B_ adenosine receptor as a key mediator of inosine-induced inhibition of spontaneous activity; and (iv) identification of BK channels as effectors of inosine-induced inhibition of SA. Together, these findings suggest that acute administration of inosine attenuates neurogenic DO via an A_2B_ adenosine receptor–BK channel-mediated pathway.

Our previous study showed that chronic treatment with inosine led to attenuation of detrusor overactivity, and was associated with alteration in mediators of sensory neurotransmission^4^. Here we describe acute, local activity of inosine in the bladder and define a molecular pathway mediating its inhibitory activity *in vitro*, that is likely to be relevant *in vivo.* Comparison of the activity of inosine on muscle strips with and without mucosa revealed a comparable reduction of spontaneous activity over a similar dose range. While we cannot rule out the possibility that inosine promotes the release of urothelial factors that influence spontaneous activity, our observations from muscle strips without mucosa demonstrate that inosine acts directly on the detrusor to reduce overactivity. Thus, inosine can act through distinct mechanisms to improve NDO under both chronic and acute treatment regimens.

Intrinsic mechanical activity generated *in vitro* is thought to underlie spontaneous contractile activity that occurs during the filling phase in various species, including humans (reviewed in ref. 17). Under pathologic conditions, increased spontaneous activity may give rise to detrusor overactivity and sensation of urgency. After spinal cord injury in rats, the pattern of spontaneous activity in the presence and absence of mucosa consisted of low amplitude/high frequency activity superimposed with regular periods of fused tetanic tension, which differed markedly from the variable low amplitude spontaneous activity observed in bladder tissue from normal animals. Although inosine reduced spontaneous activity in tissues from both control and SCI rats, the significant effect of inosine on reducing the amplified intrinsic activity evident in the context of SCI as well as attenuating non-voiding contractions, indicates the therapeutic potential of modifying local targets to ameliorate bladder overactivity.

Spontaneous activity is directly associated with modulation of resting membrane potential and generation of action potentials. This electrical activity is regulated in part by potassium channels^18^. Prior studies have reported functional interactions between adenosine receptor signalling and potassium channels in modulation of smooth muscle contractility in the coronary vasculature (reviewed in ref. 19). Using both pharmacological agents and mice with targeted deletion of adenosine receptor subtypes, it has been established that A_2A_ and A_2B_ receptors play important roles in adenosine-induced coronary artery smooth muscle relaxation and vasodilation by activation of members of the K_ATP_^20^,^21^,^22^ and K_v_^23^ channel families. Notably, adenosine failed to induce relaxation in aortic rings of A_2A_ receptor knockout mice, consistent with a dominant role for A_2A_ in smooth muscle relaxation^21^. Using a combination of pharmacological agonists and antagonists we identified A_2_ receptors as the subtypes likely to mediate the action of inosine in bladder tissues. Although NECA is considered a pan-adenosine receptor agoinst, the effect that we observed is likely due to A_2_ receptor activation: A_1_ receptor activation causes smooth muscle contraction, and NECA has been shown previously to cause relaxation in the rat bladder, an effect that is not altered by selective A_1_ receptor inhibition^24^. We observed a predominant role for A_2B_ receptor signalling in inosine-mediated attenuation of spontaneous activity in detrusor smooth muscle and identified the large conductance BK channel as the primary effector of inosine action. BK channels are among the most important regulators of detrusor smooth muscle function (reviewed in ref. 25), and alterations in either the expression or activity of these proteins have been implicated in the development of detrusor overactivity (DO). Decreased BK channel expression and function was found to correlate with increased spontaneous contractions in experimental rodent models of DO and in tissues from patients with DO^26^,^27^,^28^. Moreover, pharmacologic BK channel activation has been shown to inhibit spontaneous activity and decrease bladder tone. We observed that attenuation of spontaneous activity and reduced basal tone in detrusor strips from SCI rats by inosine was prevented in the presence of the BK channel inhibitor iberiotoxin, consistent with the idea that inosine inhibits SA by activation of BK channels.

Differences in the importance of A_2A_ and A_2B_ receptor subtypes to functional changes in various smooth muscle types may reflect differences in relative receptor expression. Evaluation of adenosine receptor mRNA levels in rat tissues have suggested that A_2B_ is the most abundant receptor subtype in bladder tissue whereas A_2A_ is more abundant in other sites^29^,^30^. In the current study, RT-PCR analysis of mucosa and detrusor tissues from control and SCI rats demonstrated no appreciable difference in expression of A_2A_ or A_2B_ in mucosa from either control or injured animals, whereas in detrusor specimens, A_2B_ mRNA levels were higher than A_2A_ levels. These findings provide a potential explanation for the identification of A_2B_ as the principal mediator of inosine activity in the bladder.

Our findings implicate A_2B_ in mediating the beneficial inhibitory effect of inosine on spontaneous activity in tissues from rats with NDO. Interestingly, a recent report implicated A_2B_ in adenosine-mediated relaxation of precontracted detrusor strips from the rat bladder^31^. Together with observations that decreases in expression of the A_2B_ receptor have been reported to contribute to a reduction in evoked detrusor relaxation with aging and with declining estrogens^30^,^32^, these findings emphasise the potential for inosine as a pharmacologic agonist of A_2B_ as well as a strategy for modulating bladder activity.

In addition to its receptor-mediated activity, inosine is known to act through a receptor-independent pathway following facilitated diffusion into target cells, mediated by members of the equilibrative nucleotide transporter (ENT) family^33^. In that study, Benowitz and colleagues demonstrated that inosine could stimulate axon outgrowth via intracellular activation of a purine-sensitive kinase^33^ subsequently identified as Mst3b^34^. We ruled out a role for direct intracellular action of inosine in attenuating spontaneous activity in bladder strips from SCI rats. The ENT1 inhibitor, NBMPR did not prevent the inosine-induced attenuation of either low or high frequency spontaneous activity (Supplementary Figure 7) suggesting that the primary action of inosine is through a receptor-dependent mechanism.

## Conclusions

In summary, this study demonstrates an important role for acute, local action of inosine in attenuation of neurogenic detrusor overactivity. Inosine inhibits spontaneous activity in bladder tissue from SCI rats through a mechanism mediated by A_2B_ adenosine receptors that impinges on large conductance BK channels. These findings suggest that inosine may be a novel intravesical therapeutic agent in the setting of neurogenic detrusor overactivity.

## Materials and Methods

### Ethical Approval

These studies and methods were carried out in accordance with the recommendations in the Guide for the Care and Use of Laboratory Animals of the National Institutes of Health. All experimental protocols were approved by the Animal Care and Use Committee of Boston Children’s Hospital (protocol #13-09-2501 R/16-08-3256 R).

### Creation of spinal cord injury

Male Sprague Dawley rats (7 wk, ~250 g, Charles River Laboratories, Wilmington, MA) were subjected to mid-thoracic spinal cord transection essentially as described^15^. Briefly, under isoflurane anesthesia a dorsal midline incision was made over the thoracic spinal cord. Superficial and deep muscle layers were incised in the midline to expose the spine and a laminectomy was performed. The dura was transected sharply and the cord was severed at T8. Gelfoam (Ethicon^TM^) was placed between the two cut ends of the spinal cord to aid in haemostasis; the dura was not closed. The paraspinal muscles and skin were closed in two separate layers. Post-operative pain was managed with meloxicam analgesia (5 mg/ml, subcutaneously (s.c). every 24 h for 3 d). Rats received Baytril antibiotic prophylaxis (100 mg/ml at 7.5 mg/kg, s.c) during the post-operative period. During the period characterised by bladder areflexia or spinal shock, bladders were emptied every 12 h by manual expression until reflex voiding returned. This period lasted for 10 d to 2 wk. Rats were monitored and weighed on a daily basis. At 6.5 weeks, cohorts of rats were subjected to either *in vivo* cystometry or bladder contractility testing. Selected tissues were also used for molecular analysis.

### *In vivo* cystometry: Intravesical delivery of inosine

The storage and emptying abilities of the bladder were assessed 6.5 wk after SCI in a subset of rats (n = 6) using conscious cystometry, essentially as described^15^. Under isoflurane anesthesia, 4 d prior to cystometry, an intravesical catheter was placed through a midline laparotomy. PE-50 tubing (Intramedic, Sparks, MD) with a flared tip was tunnelled through a dorsal incision between the scapulae into the peritoneal cavity. The tubing was introduced through the dome of the bladder and secured with a purse-string suture (6-0 prolene). The exteriorised PE-50 tubing between the scapulae was attached to a Luer lock adaptor and secured under the skin with a 2-0 silk suture. Post-operative pain was managed with meloxicam analgesia (5 mg/ml, s.c. every 24 h for 3 d). Animals also received Baytril antibiotic prophylaxis (100 mg/ml at 7.5 mg/kg, s.c.) during the 3 d post-operative period. Conscious cystometry was conducted 4 d after placement of the catheter. The catheter was attached to a physiological pressure transducer (MLT844, ADInstruments, Colorado Springs, CO) to allow measurement of intravesical pressure. The bladder was continuously infused with sterile PBS at 100 μl/min. After an equilibration period of 30–45 min, intravesical pressures were monitored over 2–3 voiding cycles with infusion of PBS (baseline). The post-void residual volume was then drained from the bladder by gravity through the PE-50 tubing. Inosine (200 μl, final concentration 1 mM dissolved in PBS) was administered intravesically through the PE-50 tubing and incubated for 15 min. Another 2-3 voiding cycles were generated with inosine as the infusate. After the last voiding cycle, post-void residual volume was measured. Voided volumes were determined by collection of voided bladder contents in a weighing scale placed below the housing cage. Bladder pressures and voided volumes were converted to digital signals using a PowerLab data acquisition system and analysed using LabChart Pro software. In this analysis, a spontaneous non-voiding contraction (SNVC) was defined as a rise in intravesical pressure greater than 5 cm H_2_O (in the absence of motion artifact or abdominal straining) that did not result in a void. Bladder capacity was defined as the sum of the voided volume and post void residual volume. Voiding efficiency was defined as the percentage of the bladder capacity that was voided. Data from 2–3 voiding cycles per condition were averaged from each animal and included analysis from both the storage and emptying phases. The amplitude and frequency of non-voiding contractions, compliance, micturition interval, voided volumes, voiding pressure (mid-void), and maximum micturition pressure were determined. At the end of analysis, rats were euthanised via CO_2_ inhalation.

### *Ex vivo* contractility studies

At 6 wk after transection, bladders from a cohort of SCI-injured animals (n = 50) were harvested following euthanasia via CO_2_ inhalation and preserved in ice-cold Kreb’s buffer (NaCI 120 mM: KCI 5.9 mM; NaHCO_3_ 25 mM; Na_2_H_2_PO_4_ 1.2 mM; MgCI.6H_2_O 1.2 mM; CaCI_2_ 2.5 mM; dextrose 11.5 mM) for *ex vivo* contractility analyses. The bladder was opened longitudinally and its base removed by cutting above the ureteral orifices. Tissue strips with intact mucosa were cut from the remaining bladder segment, mounted in tissue chambers maintained at 37 °C and bubbled with a mixture of 95% O_2_ and 5% CO_2_. Bladder tissue was attached to a force transducer (Grass Instruments) and stretched to 1.5 grams. Following an equilibration period of at least 45 min, the functional effects of pharmacological agents (inosine, adenosine receptor antagonists or agonists, potassium channel antagonists) on spontaneous activity were measured. Concentrations of pharmacological agents were either determined empirically or derived from the literature. Data were expressed as force (mN) normalised by tissue cross-sectional area (calculated from tissue weight and length measured at the end of the experiment) and presented as mean ± SEM. SA after SCI is characterised by low amplitude, high frequency contractions superimposed on large amplitude phasic contractions that occur with low frequency (Supplementary Figure 1A). These two components were analysed separately by measuring the peak-valley amplitude and peak-peak frequency after high-pass and low-pass filtering, respectively. Tissue not required for functional analysis was separated into mucosa alone and detrusor without mucosa and were flash frozen and stored at −80 °C for molecular analysis.

### RT-PCR

RNA was isolated from mucosa alone and from bladder strips without mucosa (n = 4 controls, n = 9 SCI) using TRIzol reagent along with the RNeasy MiniKit (Qiagen). RNA was reverse-transcribed using a high-capacity cDNA synthesis kit (Applied Biosystems-Life Technologies, Foster City, CA) according to the manufacturer’s instructions. cDNAs were amplified using gene-specific assays for A_2A_ and A_2B_, and relative mRNA levels were determined following normalisation to GAPDH.

### Statistical analysis

For cystometry, parameters from each animal were averaged over 3 voiding cycles at baseline and in the presence of inosine. These included the amplitude and frequency of non-voiding contractions, compliance, micturition interval, voided volumes, voiding pressure, and maximum pressure. Means ± SEM calculated from all animals under baseline conditions were compared to those following inosine treatment using a paired t-test. p < 0.05 was considered significant. For *in vitro* analysis of SA, differences in the amplitude of low and high frequency SA were compared between baseline conditions and after exposure to inosine, and between inhibitor treatment and inosine in the presence of inhibitor, using paired Student’s t-test, with p < 0.05 considered signficant. The effect of inosine on untreated and antagonist-treated tissues was determined in separate tissue strips as the effects of inosine are not completely reversible. For experiments in which pharmacological agents had little effect on baseline, data were expressed as stress (force (mN)/tissue cross-sectional area) normalised by baseline responses and presented as mean ± SEM. For experiments using potassium channel inhibitors, which had a profound effect on baseline responses, data were normalised to responses with drug alone. For dose response curves, repeated measures ANOVA test was used followed by a Tukey post-hoc test.

### Reagents

Reagents were purchased from suppliers as indicated: Inosine (Sigma-Aldrich, St. Louis, MO); CGS15943, MRS3777, PSB36, PSB603, ZM241385, PSB0777, NECA, BAY60-6583, repaglinide, iberiotoxin and apamin (Tocris Bioscience, Minneapolis, MN). Inosine was dissolved in distilled water. CGS15943, MRS3777, PSB36, PSB603, ZM241385, PSB0777, NECA, BAY60-6583, iberiotoxin and apamin were dissolved in DMSO and repaglinide was dissolved in ethanol. Exposure of tissues to vehicle alone did not alter spontaneous activity (Supplementary Figure S2). Antagonists were equilibrated in the organ bath for 15 min before the addition of an agonist. The High Capacity Reverse Transcription kit, Universal Master Mix and gene expression assays for rat A_2A_ (Rn00583935_m1), A_2B_ (Rn00567697_m1) and GAPDH (Rn01775763_g1) were from Life Technologies (Foster City, CA).

## Additional Information

**How to cite this article**: Doyle, C. *et al*. Inosine attenuates spontaneous activity in the rat neurogenic bladder through an A_2B_ pathway. *Sci. Rep.* **7**, 44416; doi: 10.1038/srep44416 (2017).

**Publisher's note:** Springer Nature remains neutral with regard to jurisdictional claims in published maps and institutional affiliations.

## Supplementary Material

Supplementary Information

## Figures and Tables

**Table 1:** Urodynamics parameters in SCI cystometrograms.

Parameters	Baseline	Inosine (1 mM)
Compliance (ml/cm H_2_O)	0.95 ± 0.32	0.75 ± 0.16
Voided volume (ml)	1.45 ± 0.10	1.24 ± 0.11*
Voiding pressure (cm H_2_O)	25.11 ± 1.48	21.62 ± 1.81*
Peak micturition pressure (cm H_2_O)	42.80 ± 4.80	40.07 ± 3.68
Intercontraction interval (min)	13.10 ± 1.49	14.39 ± 1.56
Bladder capacity (ml)	2.43 ± 0.16	1.92 ± 0.13
Voiding efficiency (%)	33 ± 8	32 ± 8
Post-void residual (ml)	0.83 ± 0.26	0.59 ± 0.14

Values are expressed as mean ± SEM. *p < 0.05.
