# Supplementary material for: Inosine attenuates spontaneous activity in the rat neurogenic bladder through an A2B pathway
Source: Sci Rep. 2017 Mar 15;7:44416. doi: 10.1038/srep44416 (PMC5353659; doi:10.1038/srep44416)
Supplement: Supplementary Information [file srep44416-s1.pdf]

Inosine attenuates spontaneous activity in the rat neurogenic bladder through an A_{2B} pathway

Claire Doyle[1,2], Vivian Cristofaro[2,3,4], Bryan S. Sack[1,2], Stefan N. Lukianov[1], Mattias Schäfer[1,2], Yeun Goo Chung[1,2], Maryrose P. Sullivan[2,3,4,*], Rosalyn M. Adam[1,2,*]

Supplementary Information

Supplementary Tables

Supplementary Table S1: Raw data relevant to Figure 2:

Control	**Baseline**	**Inosine 1mM (mean ± SEM)**	**N**	**P value**
Low frequency amplitude	0.823 ± 0.187	0.496 ± 0.173	9	*
SCI				
Low frequency amplitude	2.380 ± 0.417	1.063 ± 0.187	14	**
SCI-Inosine Dose Response	**Baseline**	**Inosine (mean ± SEM)**		
Low frequency amplitude	1.991 ± 0.390	2.279 ± 0.469 (10^{-4} M)<br>1.740 ± 0.353 (3 x 10^{-4} M)<br>0.655 ± 0.172 (10^{-3} M)<br>0.364 ± 0.177 (3 x 10^{-3} M)	8	n.s.<br>n.s.<br>**<br>***
High frequency amplitude	0.368 ± 0.068	0.4279 ± 0.0938 (10^{-4} M)<br>0.3401 ± 0.0699 (3 x 10^{-4} M)<br>0.1687 ± 0.0416 (10^{-3} M)<br>0.0955 ± 0.0255 (3 x 10^{-3} M)	8	n.s.<br>n.s.<br>*<br>***

Supplementary Table S2: Raw data relevant to Figure 3.

Pan-adenosine receptor antagonist	**CGS15943**<br>**(mean ± SEM)**	**CGS15943 + Inosine**<br>**(mean ± SEM)**	**N**	**P value**
Low frequency amplitude	1.425 ± 0.7328	1.577 ± 0.6751	7	ns, p=0.4468
High frequency amplitude	0.2028 ± 0.0482	0.2362 ± 0.0879	7	ns, p=0.2037
A_1 antagonist	**PSB36**<br>**(mean ± SEM)**	**PSB36 + Inosine**<br>**(mean ± SEM)**		
Low frequency amplitude	0.5147 ± 0.1926	0.3341 ± 0.1631	5	*, p=0.0124
High frequency amplitude	0.1632 ± 0.0772	0.1405 ± 0.0072	5	*, p=0.0489

A_3 *antagonist*	**MRS3777 (mean ± SEM)**	**MRS3777 + Inosine (mean ± SEM)**		
Low frequency amplitude	0.1650 ± 0.0416	0.1160 ± 0.0349	5	**, p=0.0034
High frequency amplitude	0.0055 ± 0.0122	0.0372 ± 0.0163	5	*, p=0.0047

Supplementary Table S3: Raw data relevant to Figure 4.

A_{2A} *antagonist*	**ZM241385 (mean ± SEM)**	**ZM + Inosine (mean ± SEM)**	**N**	**P value**
Low frequency amplitude	1.575 ± 0.4220	1.892 ± 0.4610	7	ns, p=0.3658
High frequency amplitude	0.1812 ± 0.0403	0.1740 ± 0.0395	7	ns, p=0.7188
A_{2B} *antagonist*	**PSB603 (mean ± SEM)**	**PSB603 + Inosine (mean ± SEM)**		
Low frequency amplitude	1.225 ± 0.1478	1.414 ± 0.2047	7	ns, p=0.1229
High frequency amplitude	0.1208 ± 0.0208	0.1329 ± 0.0263	7	ns, p=0.7119

Supplementary Table S4: Raw data relevant to Figure 5.

A_2 *agonist*	**Baseline (mean ± SEM)**	**NECA (mean ± SEM)**	**N**	**P value**
Low frequency amplitude	1.552 ± 0.238	2.160 ± 0.550 (10^{-9} M)<br>1.787 ± 0.363 (3 x 10^{-9} M)<br>1.838 ± 0.357 (10^{-8} M)<br>1.442 ± 0.216 (3 x 10^{-8}M)<br>1.275 ± 0.283 (10^{-7}M)<br>0.736 ± 0.274 (3 x10^{-7}M)<br>0.365 ± 0.251(10^{-6}M)	**6**	ns<br>ns<br>ns<br>ns<br>ns<br>*<br>**
High frequency amplitude	0.155 ± 0.017	0.186 ± 0.015 (10^{-9}M)<br>0.205 ± 0.022 (3 x 10^{-9}M)	**6**	ns<br>ns

		0.165 ± 0.014 (10^{-8}M)<br>0.178 ± 0.020 (3 x 10^{-8}M)<br>0.112 ± 0.018 (10^{-7}M)<br>0.099 ± 0.014 (3 x 10^{-7}M)<br>0.049 ± 0.013 (10^{-6}M)		ns<br>ns<br>ns<br>ns<br>**
A_{2B} agonist	**Baseline (mean ± SEM)**	**BAY60-6583 (mean ± SEM)**		**P value**
Low frequency amplitude	1.269 ± 0.2299	0.4472 ± 0.1149 (10^{-5} M)<br>0.3456 ± 0.0088 (3 x 10^{-5} M)<br>0.2845 ± 0.0776 (10^{-4} M)	7	*<br>**<br>**
High frequency amplitude	0.1612 ± 0.0407	0.0703 ± 0.0109 (10^{-5} M)<br>0.0624 ± 0.0070 (3 x 10^{-5} M)<br>0.0581 ± 0.0092 (10^{-4} M)	7	*<br>**<br>**

Supplementary Table S5: Raw data relevant to Figure 6.

BK blocker	**IBTX (mean ±SEM)**	**IBTX + Inosine (mean ± SEM)**	**N**	**P value**
Low frequency amplitude	2.466 ± 0.4568	2.564 ± 0.5700	7	ns, p=0.6949
High frequency amplitude	0.3410 ± 0.0773	0.4895 ± 0.1692	7	ns, p=0.1754
SK3 blocker	**Apamin (mean ± SEM)**	**Apamin + Inosine (mean ± SEM)**		
Low frequency amplitude	3.423 ± 0.7775	1.992 ± 0.8724	7	*, p=0.0450
High frequency amplitude	0.2477 ± 0.0587	0.1958 ± 0.0645	7	*, p=0.0267
K_{ATP} blocker	**Repaglinide (mean ±SEM)**	**Repaglinide + Inosine (mean ± SEM)**		
Low frequency amplitude	1.534 ± 0.3774	1.136 ± 0.3387	8	*, p=0.0337
High frequency amplitude	0.2615 ± 0.0567	0.1765 ± 0.0427	8	*, p=0.0197

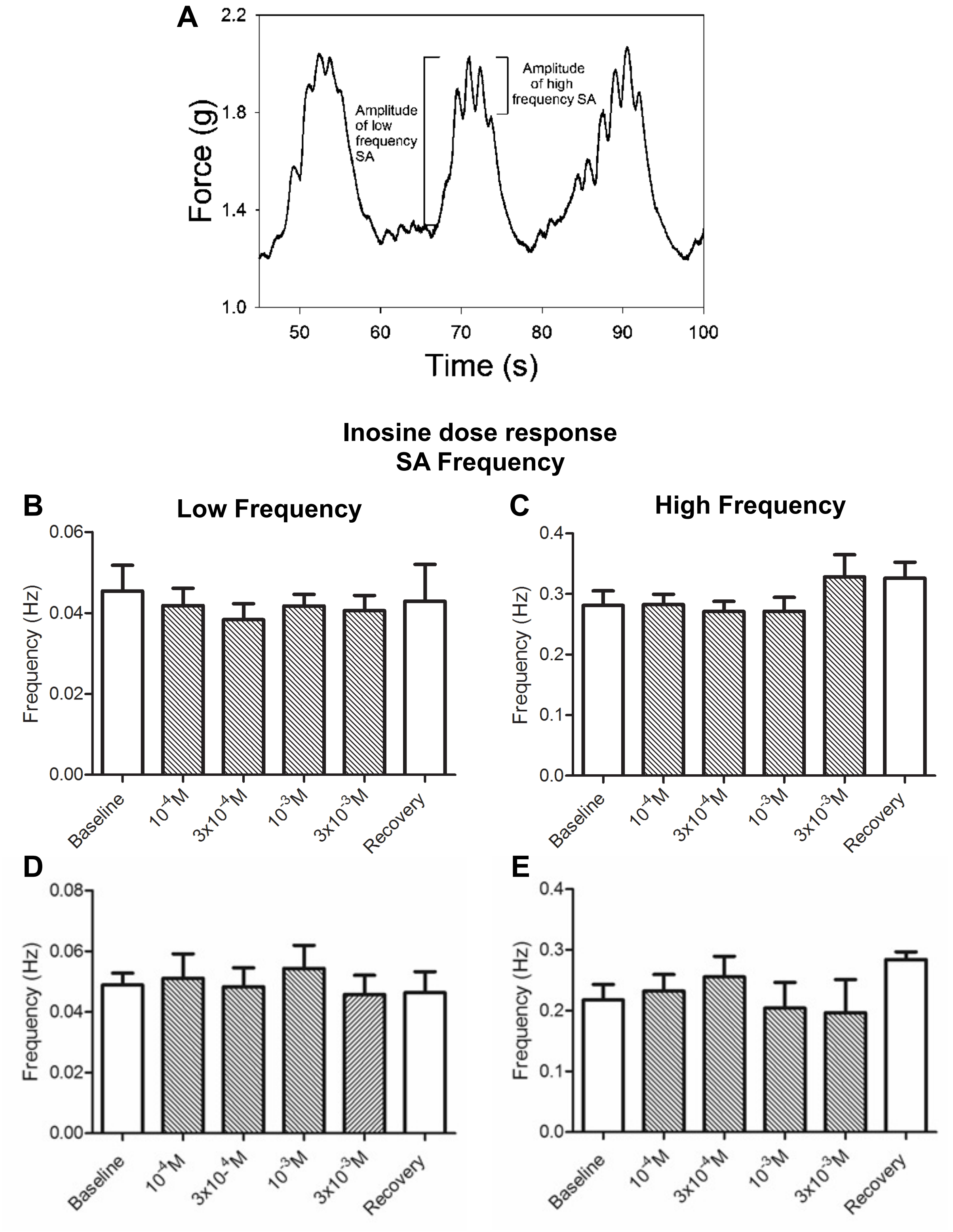

Supplementary Figure 1: Inosine does not affect frequency in SCI bladder strips with or without mucosa. (A) Trace indicating how low and high frequency SA was analysed. The amplitude of low frequency SA was determined from baseline to peak while the amplitude of high frequency SA was the additional SA present at the peak of SA. (B & C) Summary graphs indicating that inosine (10^{-4} M to 3 x 10^{-3} M) had no effect on the frequency of SA calculated from low or high frequency components in SCI bladder strips with mucosa. (D & E) Inosine did not affect frequency in SCI bladders strips without mucosa. Data are presented as mean ± SEM.

Supplementary Figure 1

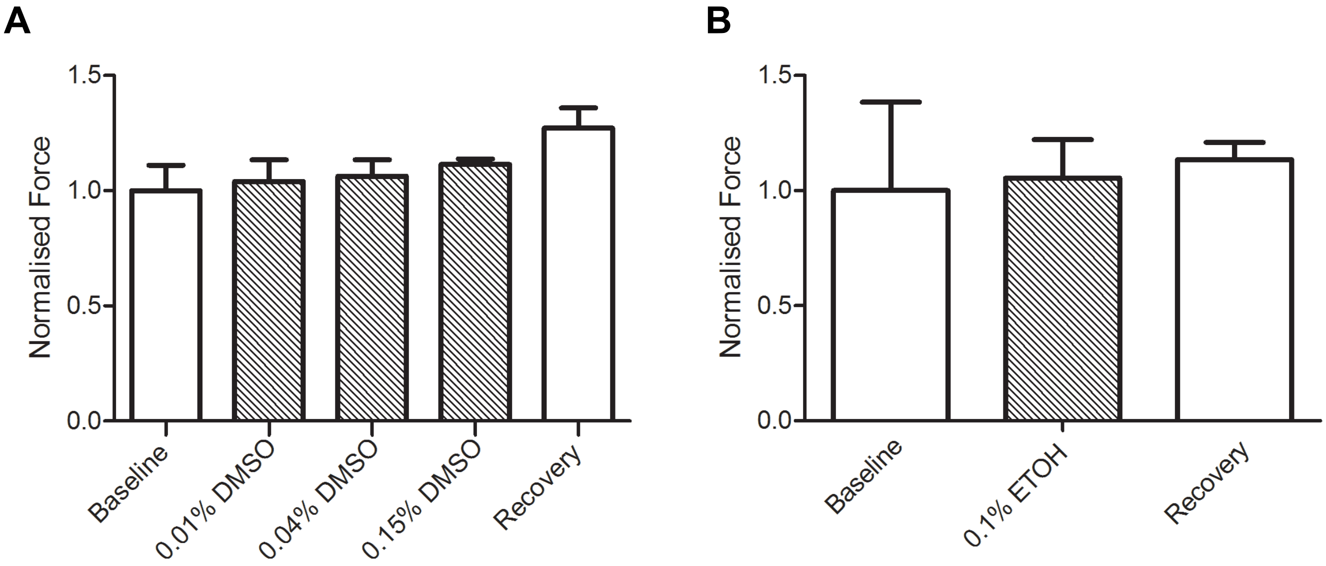

Supplementary Figure 2: Lack of effect of vehicle on spontaneous activity (A, B) Summary graphs verify the lack of effect of DMSO (indicating cumulative dose) or ethanol on amplitude and frequency (not shown) of spontaneous activity. Data are presented as mean ± SEM.

Supplementary Figure 2

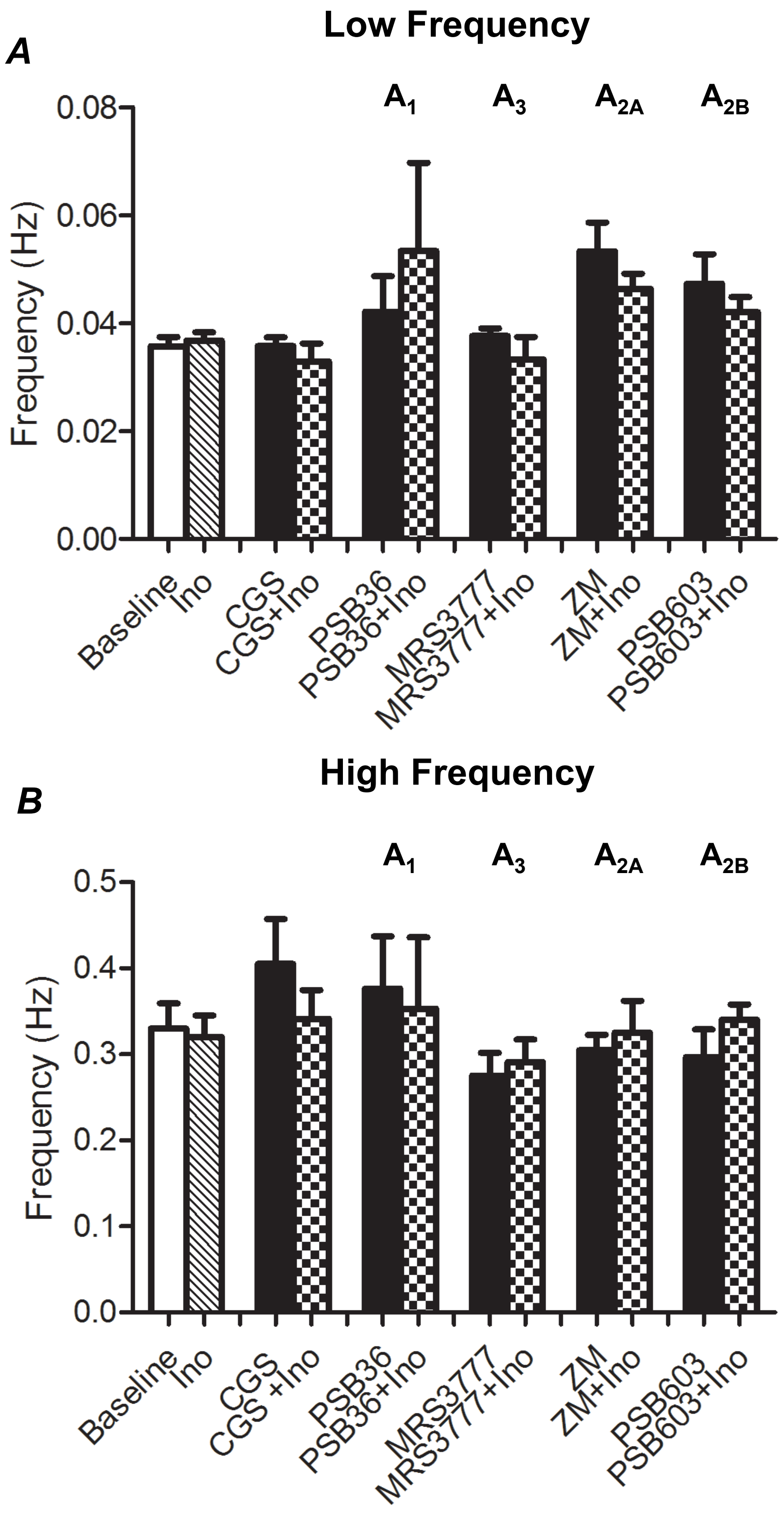

Supplementary Figure 3: Inosine and adenosine receptor antagonists do not alter the frequency of spontaneous activity. (A, B) Summary graphs illustrating that inosine (1 mM, n = 14), the pan-adenosine receptor antagonist CGS15943 (5 μM, n = 7), the A_1 antagonist PSB36 (0.1 μM, n = 5), the A_3 antagonist MRS3777 (0.1 μM, n = 5), the A_{2A} antagonist ZM241385 (100 μM, n = 7) or the A_{2B} antagonist PSB603 (0.1 μM, n = 7) had no effect on the frequency of SA at low (A) or high (B) frequency ranges. Data are presented as mean ± SEM.

Supplementary Figure 3

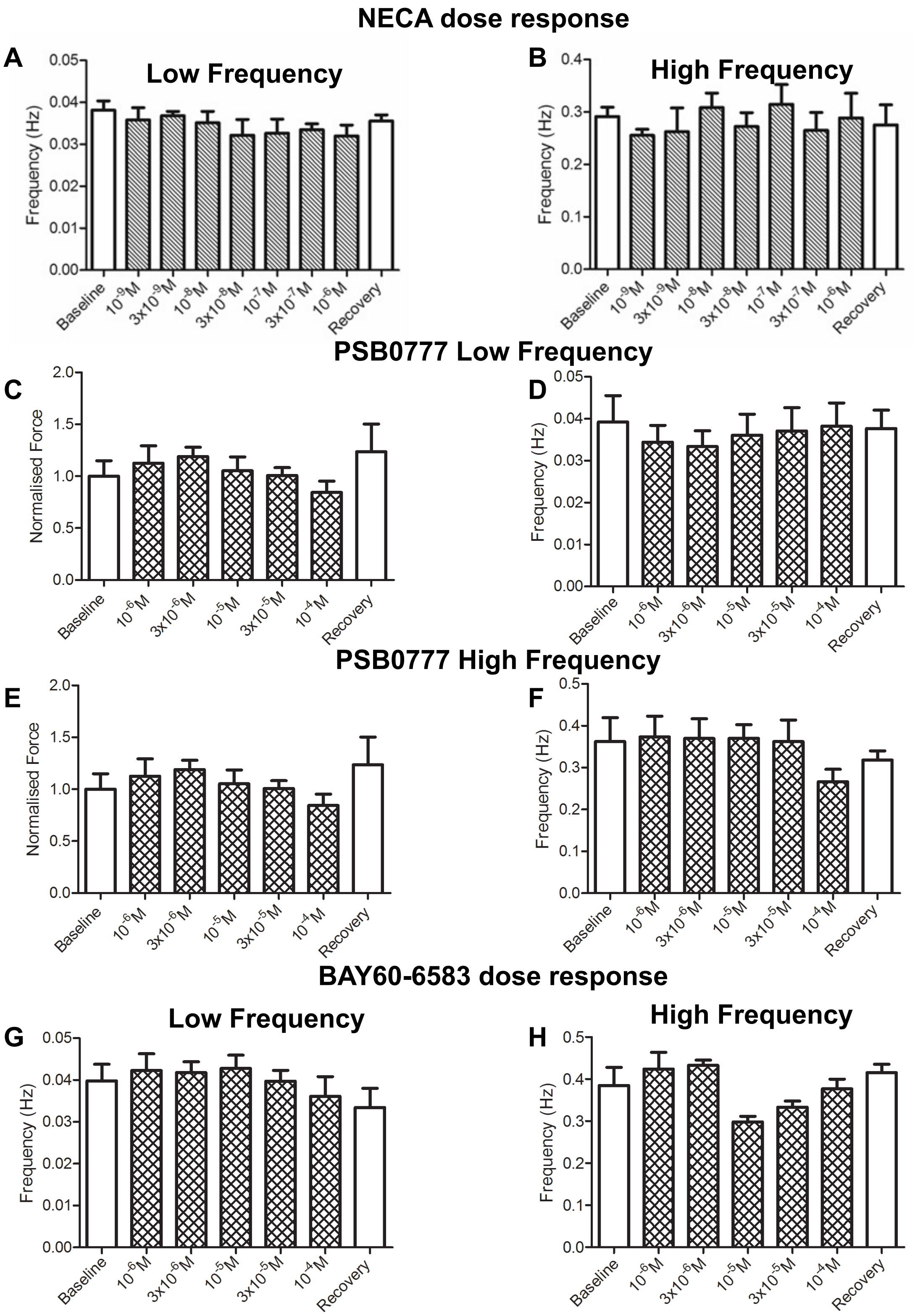

Supplementary Figure 4: Adenosine receptor agonists did not affect the frequency of SA. (A & B) Summary graphs indicating that the pan A_2 agonist NECA (10^{-9}M to 10^{-6}M, n=6) did not affect frequency of SA at low or high frequency ranges. (C-F) Summary graphs demonstrating that the A_{2A} agonist PSB0777 (10^{-6} M to 10^{-4} M, n=5) did not affect the SA amplitude (C & D) or the frequency of low or high frequency components of SA (E & F). (G & H) The A_{2B} agonist BAY60-6583 (10^{-6} M to 10^{-4}M, n=7) did not alter frequency of the low or high frequency components of SA. Data are presented as mean ± SEM.

Supplementary Figure 4

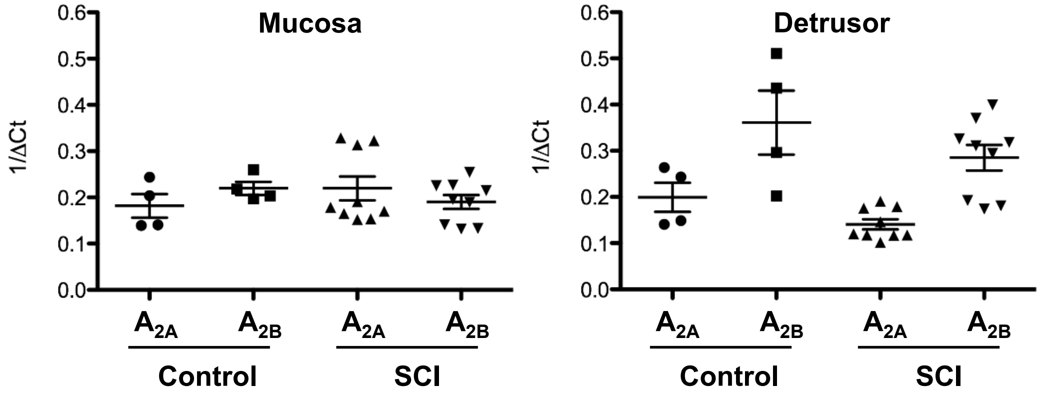

Supplementary Figure 5: A_2 receptor expression in control and SCI rat bladder tissue. RT-PCR analysis demonstrated no difference in A_{2A} or A_{2B} mRNA levels in mucosa from control or SCI rats. In bladder strips from which mucosa was removed, levels of A_{2B} were higher than those of A_{2A} in both control and SCI rats, but levels of both receptors were similar between tissues from control and SCI rats. Data are presented as mean ± SD of 1/ΔCt values.

Supplementary Figure 5

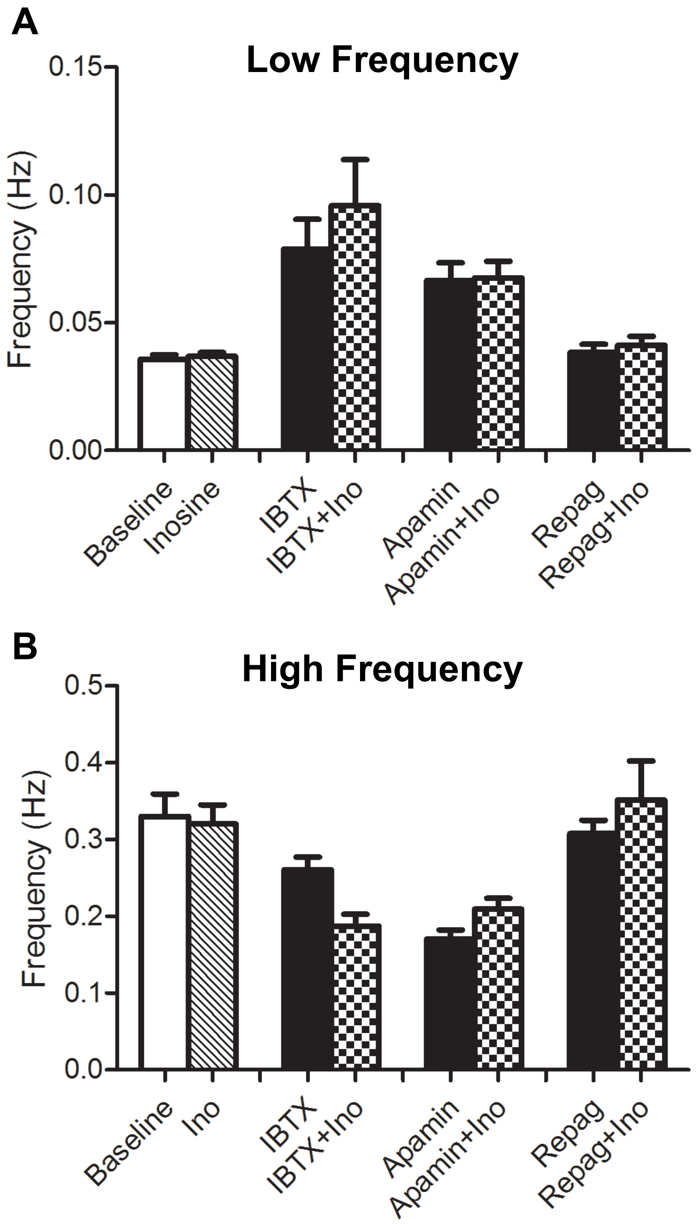

Supplementary Figure 6: The frequency of SA after blockade of K^+ channels is not altered by inosine. In the presence of either BK channel inhibitor IBTX (100 nM, n=7), SK3 channel inhibitor apamin (100 nM, n=7), or the K_{ATP} channel blocker repaglinide (10 μM, n=8), the low and high frequency components of SA were not altered by inosine. Data are presented as mean ± SEM.

Supplementary Figure 6

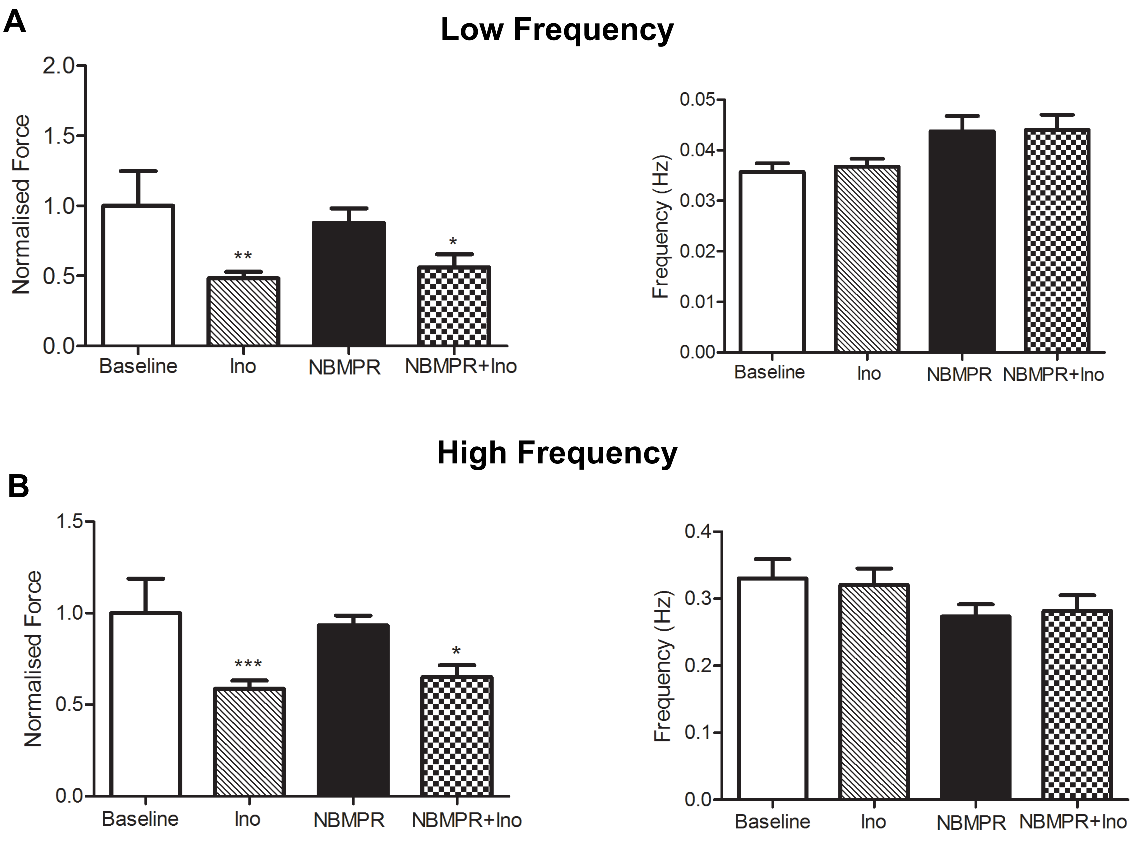

Supplementary Figure 7: Inosine does not elicit its effects through an ENT1-mediated pathway. (A) The amplitude of SA at both low (A) and high (B) frequency was significantly reduced by inosine (1 mM; n=14). However, the inhibitory effect of inosine on SA was not blocked by the ENT1 specific inhibitor NBMPR (10 µM, n=10). Inosine did not affect the frequency of SA under baseline conditions or after NBMPR treatment (right panels). Data are presented as mean ± SEM.

Supplementary Figure 7
